# Supplementary material for: Effectiveness of interactive dashboards as audit and feedback tools in primary care: A systematic review
Source: PLoS One. 2025 Jun 27;20(6):e0327350. doi: 10.1371/journal.pone.0327350 (PMC12204514; doi:10.1371/journal.pone.0327350)
Supplement: S2 Tables — (DOCX) [file pone.0327350.s002.docx]

### S2 Tables: Search strategies

**MEDLINE® (via Ovid)**

| No. | Query | Results |
| --- | --- | --- |
| 1 | exp General Practice/ or exp General Practitioners/ or exp Primary Health Care/ or Physicians/ or Professional Practice/ | 394662 |
| 2 | ((primary adj3 (care or healthcare or health-care or healthservice or healthservice)) or (practice adj3 (family or medical or general or clinical or professional)) or (family adj3 (doctor* or physician* or practitioner* or medicine)) or (general adj3 (doctor* or physician* or practitioner* or clinician*))).ti,ab. | 619917 |
| 3 | 1 or 2 | 868565 |
| 4 | Clinical Audit/ or Medical Audit/ or exp Feedback/ | 54421 |
| 5 | (((clinical or medical or plan or quality) adj3 audit*) or feed-back* or feedback*).ti,ab. | 203034 |
| 6 | 4 or 5 | 231898 |
| 7 | exp Decision Support Systems, Clinical/ or Computer Graphics/ or exp Data Visualization/ or Electronic Health Records/ or Medical Records Systems, Computerized/ or User-Computer Interface/ | 104862 |
| 8 | (dashboard or visualization* or visualisation* or computer-graphic* or ((decisionmaking or decision-support or management-support) adj3 (application* or computer* or tool* or system* or technology)) or ((electronic or computerised or computerized or digital*) adj3 (feedback* or healthrecord* or health-record* or medical-record* or patient-record* or patient-data or healthdata or health-data or healthcare or healthcare)) or (interactive adj3 (application* or computer-interface* or user-interface* or web-portal* or web-based or tool*)) or (visual adj3 (analytic* or analys*)) or (automat* adj3 (collect* or extract*))).ti,ab. | 240036 |
| 9 | 7 or 8 | 310255 |
| 10 | 3 and 6 and 9 | 1488 |
| 11 | 10 not (animals not humans).sh. | 1487 |

**Embase®**

| No. | Query | Results |
| --- | --- | --- |
| #11 | #10 NOT (('animal'/de OR 'animal experiment'/exp OR 'nonhuman'/de) NOT ('human'/exp OR 'human experiment'/de)) | 3582 |
| #10 | #3 AND #6 AND #9 | 3589 |
| #9 | #7 OR #8 | 470401 |
| #8 | dashboard:ti,ab,kw OR visualization*:ti,ab,kw OR visualisation*:ti,ab,kw OR 'computer graphic*':ti,ab,kw OR ((('decision making' OR 'decision support' OR 'management support') NEAR/3 (application* OR computer* OR tool* OR system* OR technology)):ti,ab,kw) OR (((electronic OR computerised OR computerized OR digital*) NEAR/3 (feedback* OR healthrecord* OR 'health record*' OR 'medical record*' OR 'patient record*' OR 'patient data' OR healthdata OR 'health data' OR healthcare OR 'health care')):ti,ab,kw) OR ((interactive NEAR/3 (application* OR 'computer interface*' OR 'user interface*' OR 'web portal*' OR 'web based' OR tool*)):ti,ab,kw) OR ((visual NEAR/3 (analytic* OR analys*)):ti,ab,kw) OR ((automat* NEAR/3 (collect* OR extract*)):ti,ab,kw) | 368076 |
| #7 | clinical decision support system'/exp OR 'decision support system'/de OR 'computer graphics'/exp OR 'data visualization'/exp OR 'electronic health record'/de OR 'electronic medical record'/exp OR 'electronic patient record'/exp OR 'electronic medical record system'/exp OR 'computer interface'/exp | 228441 |
| #6 | #4 OR #5 | 371126 |
| #5 | (((clinical OR medical OR plan OR quality) NEAR/3 audit*):ti,ab,kw) OR 'feed back*':ti,ab,kw OR feedback*:ti,ab,kw | 274992 |
| #4 | clinical audit'/exp OR 'feedback system'/exp | 206483 |
| #3 | #1 OR #2 | 2569497 |
| #2 | ((primary NEAR/3 (care OR healthcare OR 'health care' OR healthservice OR 'health service')):ti,ab,kw) OR ((practice NEAR/3 (family OR medical OR general OR clinical OR professional)):ti,ab,kw) OR ((family NEAR/3 (doctor* OR physician* OR practitioner* OR medicine)):ti,ab,kw) OR ((general NEAR/3 (doctor* OR physician* OR practitioner* OR clinician*)):ti,ab,kw) | 874763 |
| #1 | general practice'/exp OR 'general practitioner'/exp OR 'clinical practice'/exp OR 'primary health care'/exp OR 'physician'/de OR 'professional practice'/de OR 'medical practice'/exp | 1212082 |

**Cochrane Library**

| No. | Query | Results |
| --- | --- | --- |
| #1 | (((primary) NEAR/3 (care OR healthcare OR health-care OR healthservice OR health-service)) OR ((practice) NEAR/3 (family OR medical OR general OR clinical or professional)) OR ((family) NEAR/3 (doctor* OR physician* OR practitioner* OR medicine)) OR ((general) NEAR/3 (doctor* OR physician* OR practitioner* OR clinician*))):ti,ab,kw | 77472 |
| #2 | (((clinical OR medical OR plan OR quality) NEAR/3 (audit*)) OR feed-back* OR feedback*):ti,ab,kw | 27728 |
| #3 | (dashboard OR visualization* OR visualisation* OR computer-graphic* OR ((decision-making OR decision-support OR management-support) NEAR/3 (application* OR computer* OR tool* OR system* OR technology)) OR ((electronic OR computerised OR computerized OR digital*) NEAR/3 (feedback OR healthrecord* OR health-record* OR medical-record* OR patient-record* OR patient-data OR healthdata or health-data OR healthcare OR health-care)) OR ((interactive) NEAR/3 (application* OR computer-interface* OR user-interface* OR web-portal* OR web-based OR tool*)) OR ((visual) NEAR/3 (analytic* OR analys*)) OR ((automat*) NEAR/3 (collect* OR extract*))):ti,ab,kw | 19791 |
| #4 | #1 AND #2 AND #3 | 527 |

**Scopus**

| No. | Query | Results |
| --- | --- | --- |
| 1 | TITLE-ABS-KEY(((primary) W/3 (care OR healthcare OR health-care OR healthservice OR health-service)) OR ((practice) W/3 (family OR medical OR general OR clinical or professional)) OR ((family) W/3 (doctor* OR physician* OR practitioner* OR medicine)) OR ((general) W/3 (doctor* OR physician* OR practitioner* OR clinician*))) | 1316366 |
| 2 | TITLE-ABS-KEY(((clinical OR medical OR plan OR quality) W/3 (audit*)) OR feed-back* OR feedback*) | 905560 |
| 3 | TITLE-ABS-KEY(dashboard OR visualization* OR visualisation* OR computer-graphic* OR ((decision-making OR decision-support OR management-support) W/3 (application* OR computer* OR tool* OR system* OR technology)) OR ((electronic OR computerised OR computerized OR digital*) W/3 (feedback* OR healthrecord* OR health-record* OR medical-record* OR patient-record* OR patient-data OR healthdata or health-data OR healthcare OR health-care)) OR ((interactive) W/3 (application* OR computer-interface* OR user-interface* OR web-portal* OR web-based OR tool*)) OR ((visual) W/3 (analytic* OR analys*)) OR ((automat*) W/3 (collect* OR extract*))) | 1090085 |
| 4 | 1 AND 2 AND 3 | 2505 |

**Web of Science (Core Collection)**

| No. | Search Query | Results |
| --- | --- | --- |
| 1 | TS=(((primary) NEAR/3 (care OR healthcare OR health-care OR healthservice OR health-service)) OR ((practice) NEAR/3 (family OR medical OR general OR clinical or professional)) OR ((family) NEAR/3 (doctor* OR physician* OR practitioner* OR medicine)) OR ((general) NEAR/3 (doctor* OR physician* OR practitioner* OR clinician*))) | 701481 |
| 2 | TS=(((clinical OR medical OR plan OR quality) NEAR/3 (audit*)) OR feed-back* OR feedback*) | 649627 |
| 3 | TS=(dashboard OR visualization* OR visualisation* OR computer-graphic* OR ((decision-making OR decision-support OR management-support) NEAR/3 (application* OR computer* OR tool* OR system* OR technology)) OR ((electronic OR computerised OR computerized OR digital*) NEAR/3 (feedback* OR healthrecord* OR health-record* OR medical-record* OR patient-record* OR patient-data OR healthdata or health-data OR healthcare OR health-care)) OR ((interactive) NEAR/3 (application* OR computer-interface* OR user-interface* OR web-portal* OR web-based OR tool*)) OR ((visual) NEAR/3 (analytic* OR analys*)) OR ((automat*) NEAR/3 (collect* OR extract*))) | 603414 |
| 4 | #3 AND #2 AND #1 | 1254 |
