# Supplementary material for: Effectiveness of interactive dashboards as audit and feedback tools in primary care: A systematic review
Source: PLoS One. 2025 Jun 27;20(6):e0327350. doi: 10.1371/journal.pone.0327350 (PMC12204514; doi:10.1371/journal.pone.0327350)
Supplement: S3 Table — (DOCX) [file pone.0327350.s003.docx]

### S3 Table: Dashboard design patterns

| **Group** | **Pattern** | **Definition** |
| --- | --- | --- |
| **Data** | **Data Information** | **Patterns that identify the types of information presented and the extent of abstraction used.** |
|  | **Detailed Data Sets** | Provide the most complete view of the data, showing individual data points. |
|  | **Aggregation** | Summarizes data by combining multiple data points into a single value. |
|  | **Filtering** | Shows only specific subsets of the data based on certain criteria. |
|  | **Derived Values** | Calculated metrics or indicators derived from the original data, such as trends or averages. |
|  | **Thresholds** | Indicate specific states or values that have particular significance, such as 'good' or 'bad'. |
|  | **Single Value** | A single data point extracted from a larger data set, often the most recent value. |
| **Meta data** | **Meta Information** | **Patterns that provide context and explanation for the data presented.** |
|  | **Data Sources** | Information about where the data comes from, including the organization releasing it. |
|  | **Disclaimers** | Notes explaining the context, processing, or limitations of the data. |
|  | **Data Descriptions** | Explanations of what the data represents and what the dashboard shows. |
|  | **Update Information** | Timestamps or other indicators of when the data was last updated. |
|  | **Annotations** | Additional graphical elements added to highlight specific points, changes, or developments in the data. |
| **Visual representation** | **Visual Representation** | **Patterns that describe how the data is visually displayed.** |
|  | **Tables** | Can provide very detailed information and allow viewers to read precise values. |
|  | **Detailed Visualization** | Combined visual component including both graphs and additional informational elements, providing highly detailed information, allowing viewers to accurately read and interpret precise data values. Act as standalone components, complete with clear axis labeling, informative legends, and sufficient resolution. |
|  | **Lists** | Ordered or unordered lists of data points. |
|  | **Miniature Charts** | Small, concise visualizations without detailed axis descriptions, used for quick trend understanding. |
|  | **Gauges & Progress Bars** | Visual representations of single values within a specific context, such as progress towards a goal. |
|  | **Pictograms** | Abstract representations or symbols that illustrate data concepts. |
|  | **Trend Arrows** | Small arrows indicating the direction of change in a data value. |
|  | **Numbers** | Prominent display of individual numerical values, often used for key metrics. |
| **Page layout** | **Page Layout** | **Patterns describing how widgets are arranged on a dashboard page.** |
|  | **Open Layouts** | Widgets are placed without specific alignment rules, often in a grid. |
|  | **Stratified Layouts** | Widgets are arranged in a top-down order, often to emphasize information at the top. |
|  | **Table Layouts** | Widgets are organized into rows and columns for easy information retrieval. |
|  | **Grouped Layouts** | Widgets are grouped based on a specific relation, often labeled by a common title. |
|  | **Schematic Layouts** | Widgets are arranged in a schematic relationship, such as physical-spatial layouts. |
| **Screenspace** | **Screenspace Patterns** | **Patterns that describe how content fits onto a single screen.** |
|  | **Screenfit** | All content fits within the screen without the need for scrolling. |
|  | **Overflow** | Content exceeds the screen space, requiring scrolling. |
|  | **Detail on Demand** | Extra content is shown upon user interaction, such as mouseover or clicking. |
|  | **Parameterization** | Users can specify filters or parameters to define the information visible on the dashboard. |
|  | **Multiple Pages** | Content is split across several pages, navigable through tabs or links. |
| **Structure** | **Structure Patterns** | **Patterns describing the organization of content across multiple pages.** |
|  | **Single Page** | The dashboard consists of a single page. |
|  | **Parallel Structure** | Repeated layout and data across different sections or departments. |
|  | **Hierarchical Structure** | Gradual drill-down through multiple pages, each showing more detail. |
|  | **Open Structure** | Other types of structural relationships not captured by parallel or hierarchical structures. |
| **Interaction** | **Interaction Patterns** | **Patterns describing common interaction methods within dashboards.** |
|  | **Exploration** | Allows users to explore data elements and obtain new data views. |
|  | **Drilldown** | Users can find or focus on specific data points. |
|  | **Navigation** | Enables moving between pages or sections of a dashboard. |
|  | **Personalization** | Viewers can customize the information and layout based on personal preferences. |
| **Color** | **Color Patterns** | **Patterns describing the use of color in dashboards.** |
|  | **Shared Color Schemes** | Consistent use of colors to group related data elements. |
|  | **Data Encoding** | Using color as a visual variable to encode data categories or scales. |
|  | **Semantic Colors** | Specific colors to indicate meanings, such as 'good' or 'bad' statuses. |
|  | **Emotive Colors** | Colors chosen to evoke an emotional response. |
