# Supplementary material for: Effectiveness of interactive dashboards as audit and feedback tools in primary care: A systematic review
Source: PLoS One. 2025 Jun 27;20(6):e0327350. doi: 10.1371/journal.pone.0327350 (PMC12204514; doi:10.1371/journal.pone.0327350)
Supplement: S4 Table — (DOCX) [file pone.0327350.s004.docx]

### S4 Table: Evaluation scenarios

| **Evaluation Scenario** | **Question** | **Evaluation Aim** | **Evaluation Criteria** | **Challenges** |
| --- | --- | --- | --- | --- |
| **Task Performance (TP)** | *How does the use of the dashboard influence expected task outcomes in the dashboard’s intended task?* | To evaluate how effectively a dashboard supports the completion of specific tasks. | Accuracy, speed, and efficiency in task completion. | Identifying relevant tasks that reflect real-world use. |
| **Behavior Change (BC)** | *How does the use of the dashboard induce long lasting behavior changes in user groups?* | To assess the long-term impact of the dashboard on user behavior. | Sustained improvements (qualitative) over time. | Isolating the effects of the dashboard from other influencing factors. |
| **Interaction Workflow (IW)** | *How intuitive is the dashboard to use when executing common interaction patterns and analysis tasks?* | To evaluate the intuitiveness and efficiency of the user's interaction with the dashboard. | Integration into existing workflows, minimizing disruptions, supporting user needs. | Reflecting real-world conditions and user variability. |
| **Perceived Engagement (PE)** | *Do users feel engaged in the interaction with the dashboard beyond the immediate task completion utility?* | To gather subjective user feedback on the dashboard’s usability and satisfaction. | User satisfaction, perceived ease of use, overall engagement. | Subjective measures may be influenced by user bias and may not correlate with actual performance improvements. |
| **Potential Utility (PU)** | *How much potential does the system have for integrating useful future functions and features?* | To assess the dashboard's potential for integrating useful future functions and features. | Flexibility, extensibility, support for secondary tasks and additional functionalities. | Differentiating between current shortcomings and true potential. |
| **Algorithm Performance (AP)** | *Does the algorithm have accurate and efficient outputs?* | To evaluate the accuracy and efficiency of the algorithms embedded in the dashboard. | Precision, recall, effectiveness of the algorithms in supporting decision-making. | Ensuring algorithms are tested with relevant data and conditions. |
| **System Implementation (SI)** | *Does the implementation of the system fit its working environment?* | To assess the dashboard's integration and functionality within its intended environment. | Compatibility, stability, user training, integration within existing infrastructure. | Implementation issues from technical limitations and user resistance. |
