# Supplementary material for: Effectiveness of interactive dashboards as audit and feedback tools in primary care: A systematic review
Source: PLoS One. 2025 Jun 27;20(6):e0327350. doi: 10.1371/journal.pone.0327350 (PMC12204514; doi:10.1371/journal.pone.0327350)
Supplement: S6 Table — (DOCX) [file pone.0327350.s006.docx]

### S6 Table: Additional characteristics of included studies

| **Author, Year** | **Dashboard  Implementation** | **Data Sources** | **Time Period of Dashboard Updates** | **Benchmarking and Comparators** | **Dashboard Usage** | **Funding Sources** |
| --- | --- | --- | --- | --- | --- | --- |
| de Lusignan [1], 2021 | additional program (no connection to EHR) | UK primary care data (routinely collected and computerised) | weekly updates | practice performance against all study practices against all network practices | n.r. | Primary Care Diabetes Europe (PCDE) |
| Dreischulte [2], 2016 | Web-based portal | EHR | weekly updates | practice performance against another practice | n.r. | Scottish Government Chief Scientist Office |
| Guldberg [3], 2011 | additional program (no connection to EHR) | register data from regional or central databases | 3 updates (at month 1, 3 and 12) | each clinic performance against other participating clinics | n.r. | The Vejle CountyQuality Committee, The Central Region Denmark Quality Committee and The Danish Council for Independent Research, as well as The Tryg Foundation, Vissings Foundation, Danielsens Foundation and The A. P.Moellers Foundation Promoting Medical Science |
| Jones [4], 2023 | access via EHR | EHR | n.r. | practice performance against aggregated data from all practices in the study | n.r. | Macedon Ranges and North West Melbourne Medicare Local, Better Care Victoria, Western Health Foundation |
| Linder [5], 2010 | access via EHR | EHR | monthly updates | clinician’s performance against clinic peers against national benchmarks | 72 (28%) clinicians in the intervention group used the ARI Quality Dashboard at least once. Of these clinicians, 47 used it only once, 9 used it twice, 6 used it 3 times, and 10 used it 4 or more times (max. 7 times). | Agency for Healthcare Research and Quality and the National Heart, Lung, and Blood Institute |
| Peiris [6], 2015 | Web-based portal | EHR | usually bi-monthly updates (after uploading data to the web-based portal) | peer-ranked performance data benchmarked against other participating trial sites | 27/30 intervention sites used the audit tool to conduct data extractions and submissions to the Web portal ≥50% of the time (ie, on average data were submitted at least bimonthly). | National Health and Medical Research Council of Australia and the New South Wales Department of Health |

### References

1. de Lusignan S, Hinton W, Seidu S, Mathew M, Feher MD, Munro N, et al. Dashboards to reduce inappropriate prescribing of metformin and aspirin: A quality assurance programme in a primary care sentinel network. Prim Care Diabetes. 2021;15(6):1075-9.

2. Dreischulte T, Donnan P, Grant A, Hapca A, McCowan C, Guthrie B. Safer Prescribing--A Trial of Education, Informatics, and Financial Incentives. N Engl J Med. 2016;374(11):1053-64.

3. Guldberg TL, Vedsted P, Kristensen JK, Lauritzen T. Improved quality of Type 2 diabetes care following electronic feedback of treatment status to general practitioners: a cluster randomized controlled trial. Diabet Med. 2011;28(3):325-32.

4. Jones JL, Simons K, Manski-Nankervis JA, Lumsden NG, Fernando S, de Courten MP, et al. Chronic disease IMPACT (chronic disease early detection and improved management in primary care project): An Australian stepped wedge cluster randomised trial. Digit Health. 2023;9:20552076231194948.

5. Linder JA, Schnipper JL, Tsurikova R, Yu DT, Volk LA, Melnikas AJ, et al. Electronic health record feedback to improve antibiotic prescribing for acute respiratory infections. Am J Manag Care. 2010;16(12 Suppl HIT):e311-9.

6. Peiris D, Usherwood T, Panaretto K, Harris M, Hunt J, Redfern J, et al. Effect of a computer-guided, quality improvement program for cardiovascular disease risk management in primary health care: the treatment of cardiovascular risk using electronic decision support cluster-randomized trial. Circ Cardiovasc Qual Outcomes. 2015;8(1):87-95.
