# Supplementary material for: Effectiveness of interactive dashboards as audit and feedback tools in primary care: A systematic review
Source: PLoS One. 2025 Jun 27;20(6):e0327350. doi: 10.1371/journal.pone.0327350 (PMC12204514; doi:10.1371/journal.pone.0327350)
Supplement: S8 Table — (DOCX) [file pone.0327350.s008.docx]

### S7 Table: Quality of evidence (GRADE)

| **GRADE domain** | **Judgement** | **Concerns about certainty domains** |
| --- | --- | --- |
| Methodological limitations of the studies | All three studies [1-3] assessed with RoB2 for cluster randomized trials were found to have at least one concern and were therefore judged to have serious methodological limitations. | Serious |
| Indirectness | There are no differences in settings (all in primary care practices). The interventions in two studies [2, 3] were multifaceted. However, the studies analyzed patients with or at risk of different chronic diseases. | Serious |
| Imprecision | The results of the extracted outcomes (quality indicators) showed small effect estimates, but mostly with rather small confidence intervals. The total number of patients included was very high. | Not serious, borderline |
| Inconsistency | The direction of effects was mostly favorable for the intervention, however the magnitude of effects was mostly rather small. | Not serious, borderline |
| Publication Bias | Search methods for studies were systematic and comprehensive. Further, negative and positive outcomes were published. We therefore do not strongly suspect publication bias. | Not suspected |
| **Overall Certainty of Evidence** | **Dashboards may improve the quality of care, but it is difficult to analyze the role of the dashboards alone in multifaceted interventions. Further, it is important to consider the large number and slightly different definitions of quality indicators in primary care.** | **Low** ⊕⊕OO |

Source: Rating the certainty in evidence in the absence of a single estimate of effect[4].

### References:

1. Guldberg TL, Vedsted P, Kristensen JK, Lauritzen T. Improved quality of Type 2 diabetes care following electronic feedback of treatment status to general practitioners: a cluster randomized controlled trial. Diabet Med. 2011;28(3):325-32.

2. Peiris D, Usherwood T, Panaretto K, Harris M, Hunt J, Redfern J, et al. Effect of a computer-guided, quality improvement program for cardiovascular disease risk management in primary health care: the treatment of cardiovascular risk using electronic decision support cluster-randomized trial. Circ Cardiovasc Qual Outcomes. 2015;8(1):87-95.

3. Jones JL, Simons K, Manski-Nankervis JA, Lumsden NG, Fernando S, de Courten MP, et al. Chronic disease IMPACT (chronic disease early detection and improved management in primary care project): An Australian stepped wedge cluster randomised trial. Digit Health. 2023;9:20552076231194948.

4. Murad MH, Mustafa RA, Schunemann HJ, Sultan S, Santesso N. Rating the certainty in evidence in the absence of a single estimate of effect. Evid Based Med. 2017;22(3):85-7.
