# Supplementary material for: Effectiveness of interactive dashboards as audit and feedback tools in primary care: A systematic review
Source: PLoS One. 2025 Jun 27;20(6):e0327350. doi: 10.1371/journal.pone.0327350 (PMC12204514; doi:10.1371/journal.pone.0327350)

### S8 Figure: Content design patterns


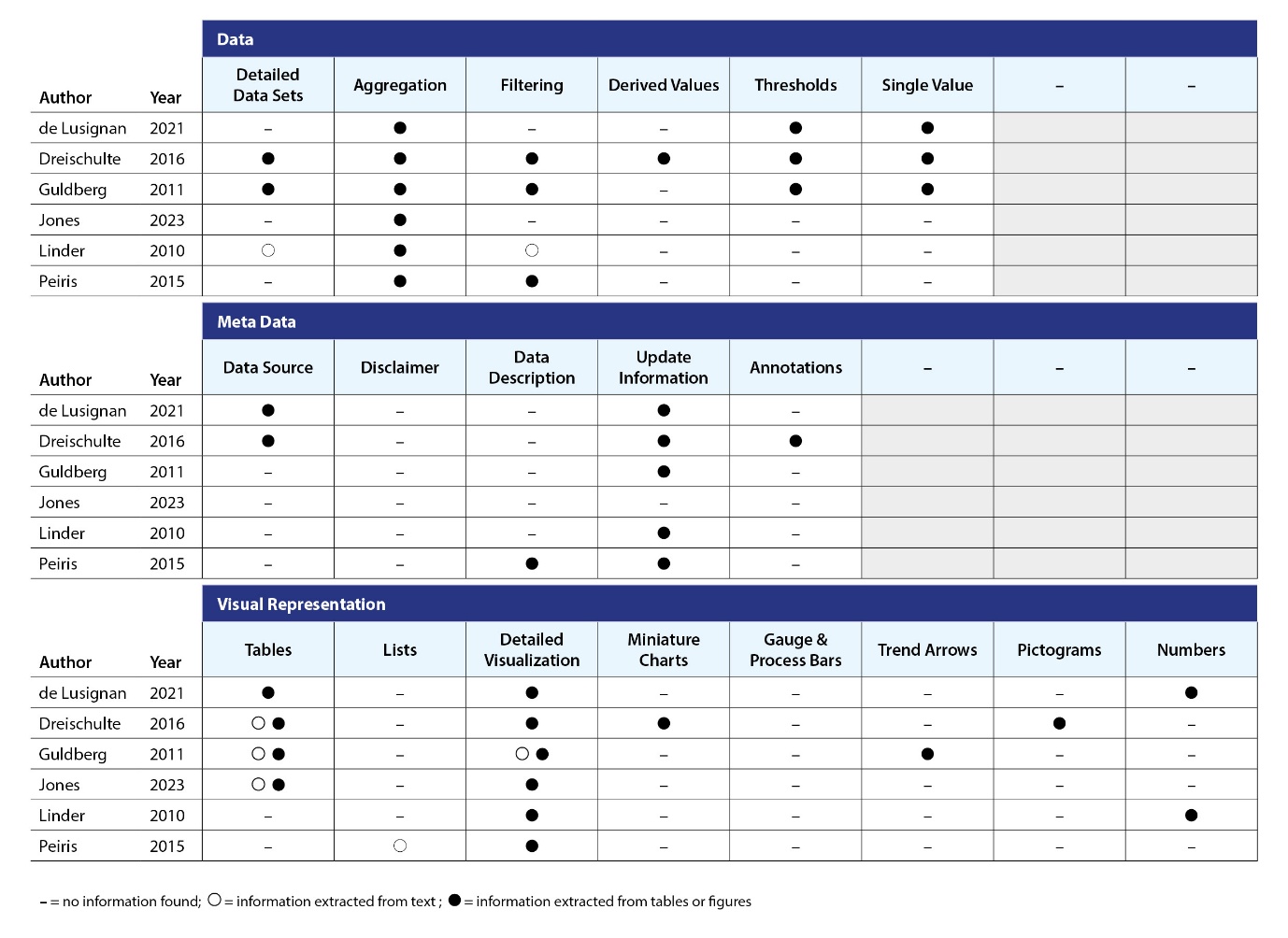


### S8 Figure: Composition design patterns


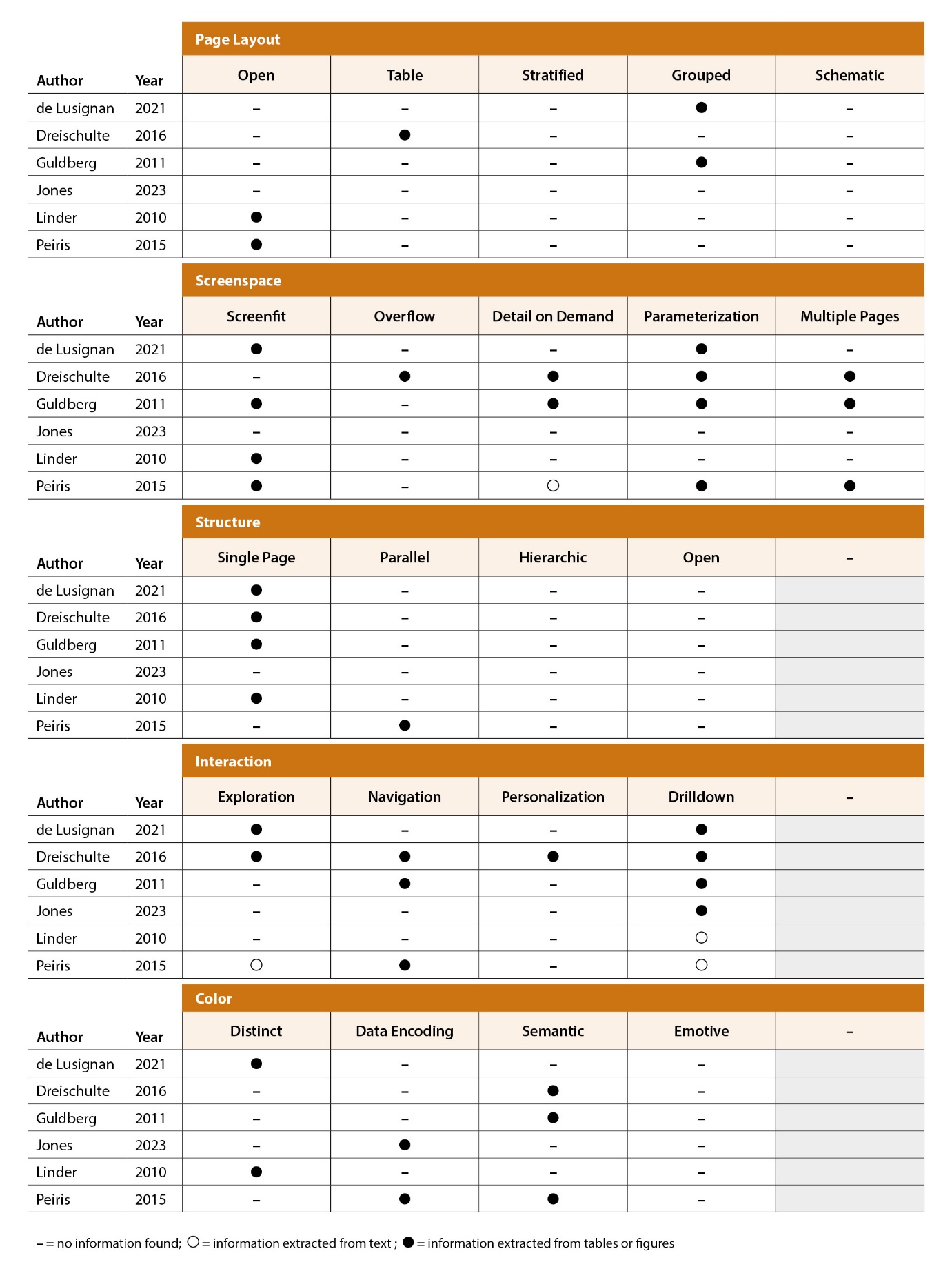


### S8 Figure: Dashboard evaluation


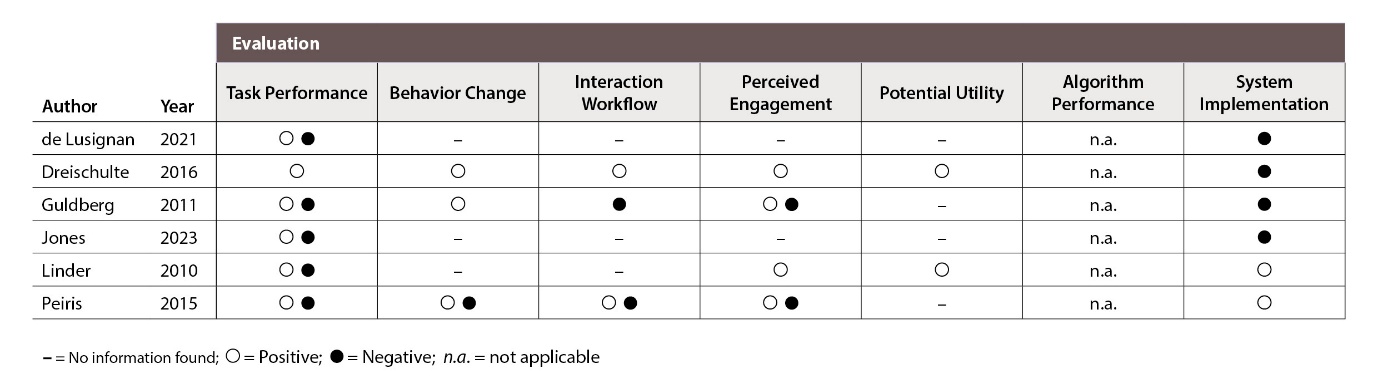

Supplement: S10 Figures — (DOCX) [file pone.0327350.s010.docx]
