# Supplementary material for: Effectiveness of interactive dashboards as audit and feedback tools in primary care: A systematic review
Source: PLoS One. 2025 Jun 27;20(6):e0327350. doi: 10.1371/journal.pone.0327350 (PMC12204514; doi:10.1371/journal.pone.0327350)
Supplement: Attachment — Submitted filename: po_rev_SR_dashboard_PTPR_V3.docx [file pone.0327350.s011.docx]

Comments to the reviews on the manuscript

**Effectiveness of interactive dashboards as audit and feedback tools in primary care: a systematic review**

June 4, 2025

We thank the reviewers and the editor for their very valuable thoughts and comments. We have improved our manuscript accordingly and now believe to provide balanced clinically relevant information for clinicians and researchers. If any other changes are needed, we are happy to include additional aspects in the manuscript.

**Journal Requirements:**

| Query | Editor’s comment | Our answer |
| --- | --- | --- |
| 1 | Please ensure that your manuscript meets PLOS ONE's style requirements, including those for file naming. The PLOS ONE style templates can be found at <https://journals.plos.org/plosone/s/file?id=wjVg/PLOSOne_formatting_sample_main_body.pdf> and <https://journals.plos.org/plosone/s/file?id=ba62/PLOSOne_formatting_sample_title_authors_affiliations.pdf> | We checked the requirements. |
| 2 | As required by our policy on Data Availability, please ensure your manuscript or supplementary information includes the following: | See 2a to 2g |
| 2a | A numbered table of all studies identified in the literature search, including those that were excluded from the analyses. | S5 Table (divided into full-text and title/abstract exclusion sheet) |
| 2b | For every excluded study, the table should list the reason(s) for exclusion. | S5 Table Column D in each sheet |
| 2c | If any of the included studies are unpublished, include a link (URL) to the primary source or detailed information about how the content can be accessed. | Not applicable. |
| 2d | A table of all data extracted from the primary research sources for the systematic review and/or meta-analysis. The table must include the following information for each study: | S9 Table |
| 2d1 | Name of data extractors and date of data extraction | S9 Table Columns B & C |
| 2d2 | Confirmation that the study was eligible to be included in the review. | S9 Table Column D |
| 2d3 | All data extracted from each study for the reported systematic review and/or meta-analysis that would be needed to replicate your analyses. | S9 Table Columns E to M |
| 2e | If data or supporting information were obtained from another source (e.g. correspondence with the author of the original research article), please provide the source of data and dates on which the data/information were obtained by your research group. | Not applicable |
| 2f | If applicable for your analysis, a table showing the completed risk of bias and quality/certainty assessments for each study or outcome. Please ensure this is provided for each domain or parameter assessed. For example, if you used the Cochrane risk-of-bias tool for randomized trials, provide answers to each of the signalling questions for each study. If you used GRADE to assess certainty of evidence, provide judgements about each of the quality of evidence factor. This should be provided for each outcome. | S7 Table for detailed risk of bias.  S8 Table for detailed quality of evidence (GRADE). |
| 2g | An explanation of how missing data were handled. | In the methods section on lines 83 to 84 it reads:  “Further, reference lists of included studies and reviews were examined to identify additional studies.”  In the limitations on lines 400 to 402 it reads:  “Despite these challenges, we are confident that our sensitive search strategy, coupled with manual screening of potentially eligible studies cited in the included articles and systematic reviews, minimized the likelihood of missing relevant studies.” |
| 3 | Please remove all personal information, ensure that the data shared are in accordance with participant consent, and re-upload a fully anonymized data set.  Note: spreadsheet columns with personal information must be removed and not hidden as all hidden columns will appear in the published file.  Additional guidance on preparing raw data for publication can be found in our Data Policy (https://journals.plos.org/plosone/s/data-availability#loc-human-research-participant-data-and-other-sensitive-data) and in the following article: http://www.bmj.com/content/340/bmj.c181.long. | We checked the requirements. |
| 4 | [Please include captions for your Supporting Information files at the end of your manuscript, and update any in-text citations to match accordingly. Please see our Supporting Information guidelines for more information: http://journals.plos.org/plosone/s/supporting-information.](http://track.editorialmanager.com/CL0/http:%2F%2Fjournals.plos.org%2Fplosone%2Fs%2Fsupporting-information./1/010f01971b560743-c82b81e1-2de5-42c1-b3a6-22e6b1a1cba7-000000/hzfOzoZ5ySs9MFgsT-n773SzD2e0O67z5f6fUtnwHLQ=213) | We added a Supporting Information content at the end of the manuscript according to the journal guidelines. |
| 5 | Please review your reference list to ensure that it is complete and correct. If you have cited papers that have been retracted, please include the rationale for doing so in the manuscript text, or remove these references and replace them with relevant current references. Any changes to the reference list should be mentioned in the rebuttal letter that accompanies your revised manuscript. If you need to cite a retracted article, indicate the article’s retracted status in the References list and also include a citation and full reference for the retraction notice. | We checked the reference list. |

**Reply to Reviewer #1:**

| Query | Reviewer’s comment | Our answer |
| --- | --- | --- |
| 1 | This paper describes the findings of a systematic review of the effectiveness of dashboards in primary care, especially in the area of audit and feedback. It describes the process and methodology very well and the results and discussion sections are clear. | We thank the reviewer for their very encouraging compliments. |
| 2 | Line 179: change ‘One studies’ to ‘One study’. | Thank you for pointing out the spelling mistake. |
| 3 | Table 2: change ‘Primary Outcome(s)’ and ‘Secondary Outcome(s)’ to ‘Primary Target(s)’ and ‘Secondary Target(s)’ to clearly differentiate these from Primary / Secondary Outcomes Results. | We changed the column headers accordingly. |
| 4 | Line 268: change ‘screenshots of the dashboards, what made the’ into ‘screenshots of the dashboards, which made the’ (it should be which, not what). | Thank you for pointing out grammatical error. |
| 5 | Line 277: What do you mean by ‘detailed visualizations’? Please give examples. | Thank you for your comment. Bach et el. (2023) defined detailed visualizations as follows (paraphrased):  “Detailed visualizations are comprehensive visual components within a dashboard that provide a high level of data detail and precision, suitable for viewers who need to read exact values or explore the data in depth.”  We interpreted visualizations as 'detailed visualizations' when they integrated graphs (e.g., bar graphs or time-series plots) together with additional contextual information (combined visual components) - such as numeric annotations, clear axis labeling, informative legends, and supplementary metrics - embedded within dashboards, providing sufficient detail to enable users to accurately read and interpret precise data values.  We added the following sentence to the manuscript on lines 282 to 283:  “[…](combined visual components including both graphs and additional informational elements) […  ]”  Unfortunately, the definition was lost in the S3 Table, therefore we added it there. Further, we added to the manuscript the following information (see also answer #7):  Lines 273 and 285: “[…] for definitions see S3 Table)”  Line 294: “[…] for definitions see S4 Table)” |
| 6 | Line 278: Why do you call gauges and arrows ‘advanced visual elements’? Please explain or add a reference. | Thank you for your comment. Unfortunately, we chose an inappropriate wording. We replaced “advanced” with “further”. |
| 7 | Line 280: Please briefly explain ‘open, table and grouped’. | In S3 Table, each dashboard design pattern is listed with a definition. We added on line 273 and 285 the following reference “for definitions see S3 Table”.  To keep the manuscript concise, we refrain from adding these definitions into the result text. Please find below the definitions extracted from S3 Table:  Open Layouts: Widgets are placed without specific alignment rules, often in a grid.  Table Layouts: Widgets are organized into rows and columns for easy information retrieval.  Grouped Layouts: Widgets are grouped based on a specific relation, often labeled by a common title. |
| 8 | Line 297: The sentence starting ‘Additionally, four studies collected mainly positive subjective feedback …’ is confusing. I presume that the feedback was positive but it could also mean that they only collected positive feedback and disregarded anything negative. Please make this clearer. Also, remove the word ‘Additionally’. | Thank you for your valuable comment. We adjusted the sentence accordingly, and it now reads as follows (lines 303 to 306):  “Four studies reported that most of the subjective feedback collected on the i-A&F dashboard’s usability and user satisfaction (“perceived engagement”) was positive. This feedback was gathered through either user involvement during development [22, 23] or post-hoc interviews [24, 25].” |

**Reply to Reviewer #2:**

| Query | Reviewer’s comment | Our answer |
| --- | --- | --- |
|  | Thank you for the opportunity to review this timely and well-conceived systematic review. The manuscript addresses a highly relevant topic within the evolving field of digital health—namely, the use of interactive dashboards as audit and feedback (A&F) tools to improve care quality in primary care settings. The study is methodologically sound, well-articulated, and offers a valuable synthesis of current evidence. It aligns with the standards of PRISMA 2020, is registered on PROSPERO (CRD42024506727), and applies appropriate tools for risk of bias (RoB 2, ROBINS-I) and quality of evidence assessment (GRADE). | We thank the reviewer for appreciating our study. |
|  | Despite its strengths, the manuscript would benefit from minor revisions to expand its discussion and address certain critical domains that were underexplored. These additions would enhance the paper’s conceptual rigor, applicability in real-world settings, and alignment with current discussions in implementation science and digital health policy. | We address these points below. |
| 1 | Strengths of the Manuscript • Clearly formulated research question with operational definitions of interactive A&F dashboards. • Broad and sensitive search strategy across five databases, executed by an information specialist. • Transparent screening, data extraction, and risk of bias assessment by independent reviewers. • Appropriately justified narrative synthesis in light of heterogeneity among studies. • Well-structured manuscript with fluent and professional English writing. • Full data availability in accordance with PLOS ONE policies. | Thank you for highlighting the strengths of our review. |
| 2 | Areas for Enhancement | We have carefully considered each point and integrated appropriate clarifications and limitations within the manuscript. However, several suggested expansions lie beyond our explicitly stated research scope—specifically, evaluating the clinical effectiveness of interactive dashboards as audit and feedback tools within primary care settings.  Nevertheless, to transparently address the points raised, we have explicitly noted relevant limitations in our revised manuscript, highlighting important aspects such as data privacy and user literacy as critical considerations for future research in digital health implementation. |
| 2.1 | Data Security, Privacy, and Anonymization The review does not address the data protection and privacy implications of using interactive dashboards in clinical settings—particularly given their reliance on electronic health record (EHR) data. There is no discussion of anonymization protocols or compliance with legal and ethical frameworks such as GDPR, HIPAA, or other relevant national data governance policies. This is a notable omission, especially considering the increasing scrutiny around patient data handling in digital health solutions. | We acknowledge that data security, privacy implications, and adherence to frameworks such as GDPR and HIPAA are critical aspects of implementing digital health solutions. The included studies don’t provide a discussion on the broader data protection and privacy implications of implementing or using interactive dashboards. Further, these aspects were beyond the scope of this systematic review, which focused explicitly on the effectiveness of interactive dashboards. Future research should include an explicit assessment of compliance and best practices in data handling (see 3.1 below). |
| 2.2 | Data Input Quality and Information Reliability The accuracy of any dashboard is only as reliable as the quality of the data it receives. The authors do not discuss the risk of poor data quality—such as incomplete, outdated, or incorrectly coded clinical records—compromising the validity of the dashboards' outputs. This is a critical limitation that should be explicitly acknowledged and discussed. | While the quality of data input into dashboards undeniably influences their utility and accuracy, assessing data quality and reliability was beyond this review’s scope.  A brief review revealed that some studies affirm that steps were taken to ensure reliability (Jones 2023), refer to previous validation efforts for key data points (Linder 2010), acknowledge how data entry methods affect data capture (Jones 2023), and link general data quality improvements to policy contexts (de Lusignan 2021). Therefore, future research should specifically evaluate the impact of data quality on dashboard effectiveness (see 3.1 below). |
| 2.3 | Digital and Health Literacy of End-Users The successful implementation of dashboards is contingent on clinicians’ ability to interpret visualizations and engage with interactive digital tools. The manuscript does not consider the heterogeneity in digital and health literacy among primary care professionals, which may act as a barrier to uptake and utilization. | We acknowledge that variability in digital and health literacy among primary care clinicians may significantly influence dashboard uptake and effectiveness. However, our review did not address this aspect explicitly, as it aimed solely at synthesizing available evidence on clinical effectiveness.  A brief review revealed that the sources do not explicitly label clinician digital or health literacy heterogeneity as a barrier using those precise terms, but they strongly suggest that factors like inadequate training and support (Linder 2010, Guldberg 2011), poor tool usability and integration (Guldberg 2011, Jones 2023), and potentially a lack of understanding regarding underlying data capture methods (Jones 2023) significantly impede the successful uptake and utilization of dashboard interventions by primary care professionals. The inclusion of training, support, and user-centric design (like co-design) in some interventions suggests an implicit recognition that user capabilities and comfort with technology are critical for success. We added the following sentence to the “Implications for practice and future research” section in the discussion (lines 395 to 396):  “[…], particularly given the heterogeneity in digital and health literacy among clinicians, which may influence uptake and successful utilization.” |
| 2.4 | Digital Inclusion and Infrastructure Readiness The included studies are drawn from high-income settings, but the manuscript does not consider variability in infrastructure—such as internet connectivity, hardware availability, and IT support—which may significantly affect dashboard implementation in low-resource environments. These contextual factors should be discussed to better inform the generalizability of the findings. | Our systematic review primarily included studies from high-income settings, limiting the generalizability of findings to low-resource environments, where infrastructure readiness may vary significantly. We agree that future reviews and primary studies should explicitly consider digital inclusion and infrastructure readiness to ensure broader applicability (see 3.1 below). |
| 2.5 | Interoperability Across Information Systems A major omission in the discussion is the lack of attention to interoperability challenges. Many health systems operate with diverse, non-integrated platforms. The feasibility and impact of dashboards are critically dependent on their ability to interoperate with existing EHRs and data repositories. Without such integration, dashboards may become redundant or underutilized. This topic is absent from the current version and should be included. | Interoperability of dashboards with existing EHR systems is critical for practical implementation and scalability. We cover this aspect with the dashboard evaluation scenario “system implementation” in the result section. We expanded the corresponding part and it now reads as follows (lines 307 to 308):  “Only two i-A&F dashboards [23, 24] were directly integrated into existing EHRs. The other four i-A&F dashboards [22, 25-27] were independent of the EHR (“system implementation”).”  Nonetheless, future research should address these aspects in detail to inform practical dashboard implementation (see 3.1 below). |
| 2.6 | Reviewer Concordance (Kappa Statistic) While the manuscript notes that two independent reviewers conducted the screening process, no measure of interrater agreement (e.g., Cohen’s kappa) is reported. Although optional under PRISMA, including a concordance metric is a recommended good practice to demonstrate methodological rigor. | We added an interrater agreement metrics to the document (methods and result section, see answer to 3.4 below) although it is not mentioned in a word either in the PRISMA checklist nor in the PRISMA statement paper nor in the PRISMA explanation and elaboration paper. |
| 3 | Recommendations for Revision | See 3.1-3.4 |
| 3.1 | Add a paragraph in the Discussion section explicitly addressing the above issues, especially data privacy, system interoperability, and infrastructure gaps. | We added the following paragraph to the “Implications for practice and future research” section in the discussion (lines 402 to 410):  “Several important implementation and contextual factors were not systematically addressed in this review, as they were outside the predefined scope and were infrequently reported in the included studies. These include data security and privacy considerations (e.g., GDPR, HIPAA compliance), data input quality and reliability, digital and health literacy among end-users, digital inclusion and infrastructure readiness in low-resource settings, and details about interoperability with existing health information systems. While these factors are critical for the real-world adoption and effectiveness of interactive dashboards in primary care, they were not the primary focus of our analysis. We recommend that future research and reporting on digital A&F tools in primary care explicitly address these domains to enhance the applicability and generalizability of findings.” |
| 3.2 | Acknowledge these limitations in the “Implications for Practice” or “Limitations” section, emphasizing the importance of digital readiness and equity. | See 3.1 |
| 3.3 | Where possible, clarify whether any of the included studies reported on actual dashboard usage analytics, user feedback, or usability testing—information that would be valuable for implementation stakeholders. | In the results section, lines 266 to 268, we discussed dashboard usage, noting that only two studies provided some information. And on lines 303 to 306, we reported information about subjective user feedback from four studies.  In the discussion section (Implications for practice and future research), we addressed the importance of involving end users in the process of dashboard developing (covering user feedback and usability testing). Therefore, we further expanded this section and added the following sentence about dashboard usage (lines 399 to 401):  “Further, future research should also establish consistent reporting standards regarding actual dashboard usage analytics to more accurately assess dashboard implementation and impact.” |
| 3.4 | Consider reporting (or explicitly noting the absence of) interrater agreement metrics during study selection. | We added to the methods section (lines 110 to 111) the following:  “Cohen’s Kappa was used to assess the agreement between the raters.”  We added to the result section (lines 167 to 168) the following:  “The reviewers (FLM and JMB) achieved interrater agreement in the screening of titles/abstracts and full texts, with respective Kappa scores of 0.84 and 0.85.” |
| 4 | This manuscript offers a valuable and methodologically sound contribution to the literature on digital quality improvement tools in primary care. The findings are relevant to practitioners, researchers, and policymakers. With the inclusion of the recommended revisions—particularly those expanding the scope of the discussion—the article will be significantly strengthened in both academic and practical terms. | Thank you for your final comment, very much appreciated. |
